# A Review of Bunyamwera, Batai, and Ngari Viruses: Understudied *Orthobunyaviruses* With Potential One Health Implications

**DOI:** 10.3389/fvets.2018.00069

**Published:** 2018-04-12

**Authors:** M. Fausta Dutuze, Manassé Nzayirambaho, Christopher N. Mores, Rebecca C. Christofferson

**Affiliations:** ^1^School of Veterinary Medicine, Louisiana State University, Baton Rouge, LA, United States; ^2^College of Agriculture and Animal Sciences and Veterinary Medicine, University of Rwanda, Kigali, Rwanda; ^3^School of Public Health, University of Rwanda, Kigali, Rwanda

**Keywords:** *Orthobunyavirus*, Bunyamwera virus, Ngari, Batai, Rwanda, arboviruses of cattle

## Abstract

Bunyamwera (BUNV), Batai (BATV), and Ngari (NRIV) are mosquito-borne viruses of the Bunyamwera serogroup in the *Orthobunyavirus* genus of the Bunyaviridae family. These three viruses have been found to cause disease in both livestock animals, avian species, and humans. Thus, these viruses pose a potential threat to human public health, animal health, and food security. This is especially the case in the developing nations, where BUNV and NRIV are found, mainly in Africa. BUNV and BATV are fairly well characterized, while NRIV is not well characterized owing to only sporadic detection in human and animal populations in Africa. Reassortment is common among bunyaviruses, but NRIV is believed to be the only natural reassortant of the Bunyamwera serogroup. It resulted from a combination of BUNV S and L segments and the BATV M segment. This indicates at least some level co-circulation of BUNV and BATV, which have no historically been reported to overlap in geographic distributions. But as these viruses are undercharacterized, there remains a gap in the understanding of how such reassortment could occur, and the consequences of such. Due to their combined wide range of hosts and vectors, geographic distributions, potential severity of associated diseases, and potential for transmissibility between vertebrate hosts, these viruses represent a significant gap in knowledge with important One Health implications. The goal of this review is to report available knowledge of and identify potential future directions for study of these viruses. As these are collectively understudied viruses, there is a relative paucity of data; however, we use available studies to discuss different perspectives in an effort to promote a better understanding of these three viruses and the public and One Health threat(s) they may pose.

## Introduction

**Figure 1:**
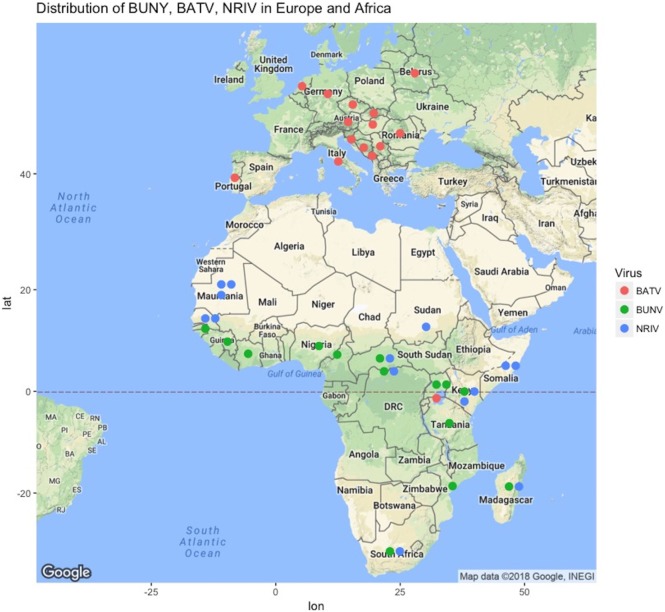
Geographic locations of reports of Bunyamwera (green dots), Ngari (blue dots), and Batai (green dots) across Europe and Africa from literature or ProMED-Mail (http://www.promedmail.org). Not shown are periodic isolations in Russia, India, Malaysia, and China, as well as Cache Valley Fever Virus in North America and the years of report, which are given in Table S1 in Supplementary Material.

Bunyamwera virus (BUNV), Batai virus (BATV), and Ngari virus (NRIV) are members of the Bunyamwera serogroup in the *Orthobunyavirus* genus of the Bunyaviridae family (1–5). They are thought to be primarily transmitted by mosquitoes. BUNV and NRIV are widely distributed throughout large parts of Africa, while BATV is found throughout Asia and Europe (Figure 1; Table S1 in Supplementary Material). The genome structure is a single-stranded, negative sense, tri-segmented RNA: small (S), medium (M), and large (L) that respectively encode the nucleocapsid, envelope glycoprotein, and polymerase protein. Additionally, the S and M segments encode non-structural proteins (NSs) (3–6). Reassortments are relatively common among Bunyaviridae family members and even within the *Orthobunyavirus* genus, but NRIV is the only naturally occurring reassortment in the Bunyamwera serogroup, though experimental reassortants have been derived (1, 7–10). Evidence suggests that NRIV arose from a natural reassortment event resulting from co-infection of BUNV and BATV, as NRIV possesses the M segment of BATV combined with the S and L segments from BUNV (1, 3, 11).

Bunyamwera virus, BATV, and NRIV are etiological agents of diseases in humans and domestic animals. The disease associated with BUNV has been reported to cause mild symptoms, such as fever, joint pain, and rash in many mammals, including humans (2, 12–14). BATV causes a mild flu-like illness in humans, is associated with a more severe disease in ruminants where it is manifested by abortions, premature births, and genetic defects (12, 15, 16). NRIV has been associated with fatal hemorrhagic fevers in both humans and ruminants (4, 17).

Bunyamwera virus is considered the prototype of the Bunyaviridae family, as it is the most characterized (18, 19). BATV is also relatively well studied given its wide distribution across Europe and Asia, while NRIV is the least studied of the three (1, 15). Studies of NRIV have been focused on genomic comparisons with other Bunyamwera group viruses (BUNV, BATV, and Ilesha viruses) and the similarity of its clinical manifestations with rift valley fever virus (RVFV) (1, 3). RVFV is an important virus of the Bunyaviridae family in the *Phlebovirus* genus, which is associated with abortions in ruminants (cattle, goats, and sheep) and mild (flu-like) to severe (hemorrhagic fever, encephalitis, and ocular troubles) symptoms in humans and ruminants. RVFV causes large outbreaks on the African continent and Arabian Peninsula and is a major public health and food security concern (4, 20–23).

Ngari virus is of particular public health relevance as it was identified as other etiologies of febrile illnesses in humans in Sudan, Somalia, and Kenya outbreaks in 1988, 1997, and 1998, respectively (1, 4, 5, 24). In these instances, molecular-based techniques, such as nucleic acid amplification and sequencing, were rarely used and diagnosis was based on clinical manifestations, thus the disease was diagnosed as the most common febrile illnesses (1, 4, 5, 24). Consequently, in the Sudanese outbreak, the disease was improperly diagnosed as malaria, as in malaria-endemic countries 70% of febrile illnesses are treated as presumptive malaria, often without proper medical examination and a laboratory analysis (25). In Somalia and Kenya, as it was during an ongoing RVFV, the disease was mistakenly taken for RVFV. Thus, the burden of disease of NRIV and likely BUNV and BATV as well is underreported and their public health impact under-appreciated.

While these viruses have heretofore been primarily identified in Europe and Asia (BATV) and Africa (BUNV and NRIV), there is a potential for worldwide spread. This is more evident as global trade and travel move people and goods around the world at records rates and continued development and urbanization bring domesticated animals and humans into greater contact with potential cryptic zoonotic transmission cycles. The two most recent importations of Old World viruses into the Western Hemisphere—chikungunya and Zika—highlight this risk (26, 27). When these viruses were introduced, millions were infected in record time, as there was no prior immunity to impede transmission through human populations. Similarly, there is not much known regarding the potential for herd health against these viruses in human and animal populations in the Western Hemisphere, though there is evidence to suggest that *Orthobunyaviruses* of the New World would offer some potential for cross-protection (28).

The aim of this review is to compile the available knowledge on these three viruses (BUNV, BATV, and NRIV) in an effort to identify potential future lines of investigation to fill any gaps. Fundamental concepts will be described and, where data permits, different perspectives discussed. Emphasis will be made on the relationships among molecular characteristics, transmission mechanisms, geographic distributions, and clinical manifestations among the three viruses, as well as with other related viruses. We performed comprehensive searches in PubMed and Google Scholar by searching for material related to BUNV, BATV, and NRIV epidemiology, vector competence, field studies/cases, and laboratory characterization studies. References relevant to the three viruses were included and exclusion of material was for redundancy (reviews, e.g.).

## Origins and Geographic Distributions of BUNV, BATV, and NRIV

Bunyamwera virus was first isolated in 1943 from *Aedes* mosquitoes collected in the Semliki Forest, Uganda as part of a yellow fever surveillance effort (13, 18, 29). BUNV is now considered endemic in several African countries, such as Uganda, Tanzania, Mozambique, Nigeria, Guinea, South Africa, and Democratic Republic of Congo (Figure 1). Recently, Cache Valley Fever virus was classified as a strain of BUNV, which expands the overall geographic range to North America, and additional strains of BUNV have been isolated in Argentina (2). A recent compilation of studies reported the presence of BUNV in Senegal, Guinea, Ivory Cost, Nigeria, Cameroon, Central African Republic, Kenya, Uganda, South Africa, and Madagascar (30).

Ngari virus was first isolated from *Aedes simpsoni* mosquitoes in 1979 in Southeastern Senegal. It was then recovered from several mosquito species in Senegal, Burkina Faso, Central African Republic and Madagascar (1988–1993) (31). The first suspicion of its potential pathogenicity in humans was when the virus was isolated from two patients in Dakar in October and November 1993 (31). In Kenya and Somalia (1998–1999), the Garissa strain of NRIV was identified in what was thought to be a RVFV outbreak, owing to the hemorrhagic manifestations of infection (4). In 2010, during an ongoing RVFV outbreak in Mauritania, NRIV was again isolated in goats (32). NRIV has additionally been isolated in Burkina Faso, Central African Republic, and Madagascar (30). In all these cases, microscopy, RT-PCR, and complete nucleotide sequences were used in the differential diagnostic panels and confirmed the differentiation between other Bunyaviruses (BUNV, BATV, ILEV, and RVFV) as well as other suspected agents (malaria) (3, 4, 32).

Batai virus is one of the most geographically widespread members of the *Orthobunyavirus* genus (Figure 1) (1, 33). It also has the most broad vector range as it was isolated from *Anopheles maculipennis* in Czechoslovakia in 1950, from *Culex gelidus* Theo mosquitoes in Malaysia, from *Anopheles barbirostris* mosquitoes in Chittoor district in India, and from *A. maculipennis* and *Aedes punctor* mosquitoes collected in what is modern Ukraine (1, 18, 34–37). In Europe, evidence for circulation exists in Norway, Sweden, Finland, Slovakia, the Czech Republic, Croatia, Serbia, Bosnia, Montenegro, Italy, Hungary, Romania, Austria, Portugal, Germany, and Belarus (38). The seroprevalence reported in humans in Europe is low: less than 1% in Sweden, Finland, Germany, Austria, and parts of the former Yugoslavia. However, there was a 32% seroprevalence reported in southern Slovakia (15). Similarly, seroprevalence in animals varied from 1 to 46% (15). Interestingly, BATV is not widely reported on the African continent, having only been reported in Uganda (1). However, the distribution of NRIV as well as the presence of suitable hosts and vector suggests that BATV might be present in other African countries and be underreported, or at least has the potential to be so.

Although no research has been conducted on the asymptomatic presentation of BUNV, BATV, and NRIV infections in vertebrate hosts, other arboviral viruses across several genera are known to present as mostly asymptomatic or as a mild influenza-like illness (39). Therefore, it is possible that NRIV, BUNV, and even BATV were circulating before successful isolation and that they may silently circulate in many areas, where no serological studies have been conducted that may detect asymptomatic or mild, generalized disease.

Ngari virus was isolated 35 years ago, nearly 30 years after the parental viruses, BUNV and BATV. Given the geographical history of BUNV and BATV, the speculation is that the first reassortment leading to new viral progeny likely resulted from the introduction of BATV into the population of already-circulating BUNV in Africa. Many factors may have contributed to the introduction of BATV in East Africa, likely long-distance bird migration (40). But these distinct geographies of BATV and BUNV also suggest that NRIV likely has developed its own niche and propogates as a distinct virus rather than relying on repeated introductions and chance co-infections of BATV and BUNV in the wild.

## Classification, Genomic Characteristics, and Replication in Host Cells

### Classification

The Bunyamwera group is one of 18 serologically defined arbovirus serogroups belonging to the *Orthobunyavirus* genus of the family Bunyaviridae (16). The 18 serogroups are *Anopheles* A, *Anopheles* B, Bakau, Bunyamwera, Bwamba, group C, Capim, California, Gamboa, Guama, Koongol, Minatitlan, Nyando, Olifanstlei, Patois, Simbu, Tete, and Turlock (41). The viruses of this genus have been classified into these different serogroups based on serological relationships of complement fixing antibodies, as well as hemagglutinating assay results and neutralizing antibodies (42).

The classification of the Bunyaviridae family has evolved, since its first organization in 1971 when the Subcommittee on Interrelationships Among Catalogued Arboviruses (SIRACA) conducted a serological study of 23 viruses. They subsequently classified 14 viruses as belonging to the Bunyamwera group viruses and further identified five subgroups or complexes: Bunyamwera, Cache Valley, Wyeomyia, Kairi, and Guaroa (43). In 1978, this classification was updated based on the antigenic relationships of Bunyamwera group viruses established by the cross-neutralization pattern of virus-specific hyperimmune antibodies (44, 45). From this effort emerged three subgroups that diverge largely over geographic origin: South America, Africa, and Europe/Asia (of which BATV is the only member) (28). While there was very little cross-neutralization among members of the South-American and African subgroups, BATV is cross-neutralized by other virus-specific antibodies from the other two subgroups (28). As of 1996, there are over 30 Bunyamwera serogroup viruses that have been identified and classified (46). As mentioned, CVV was also recently reclassified as a strain of BUNV (2). Table 1 shows the 32 Bunyamwera group viruses and the geographic areas, where they were first isolated, as well as their main vertebrate hosts and principal vectors. Most of the Bunyamwera serogroup viruses are etiological agents of human and animal diseases (5, 13, 47–49).

**Table 1:** The 32 viruses of the Bunyamwera group, geographic areas of origin, main hosts, and principal vectors, including relevant references.

Virus	Abbreviation	Source, location, year of first isolation	Main hosts	Principal arthropod vectors	Reference
AG83 1746		South America	Rodents	Mosquitoes	(46)
Anhembi	AM	Brazil	Not known	Mosquitoes (culicine)	(28, 46)
Batai	BAT	*Anopheles maculipennis*, Czechoslovakia, 1950	Humans, ruminants, sentinel animals (birds)	Mosquitoes (anopheline, culicine), Biting midges	(28, 34, 46)
BeAr 3282208		South America	Not known	Mosquitoes	(46)
Birao	BIR	*Anopheles pharoensis*, Central Afr. Rep., 1969	Not known	Mosquitoes (anopheline)	(28, 34, 46)
Bozo		Africa	Not known	Mosquitoes (anopheline, culicine)	(46)
Bunyamwera	BUN	*Aedes* spp., Uganda, 1943	Humans	Mosquitoes (anopheline, culicine)	(28, 34, 46, 50)
Cache valley	CV	*Culiseta inornata*, Utah, 1956	Sheep, cattle, marsupials	Mosquitoes (anopheline, culicine)	(28, 34, 46)
CbaAr426		South America	Not known	Mosquitoes	(46)
Fort Sherman		Human, Panama, 1985	Humans	Mosquitoes	(34, 46)
Germiston	GER	South Africa	Humans, rodents, sentinel animals	Mosquitoes (culicine)	(28, 46)
Guaroa	GRO	Colombia	Humans	Mosquitoes	(28)
Iaco		South America	Not known	Mosquitoes (culicine)	(46)
Ilesha	ILE	Human, Nigeria, 1957	Humans	Mosquitoes (anopheline)	(28, 34)
Kairi	KRI	Trinidad	Equine, primates, rodents, sentinel animals	Mosquitoes (culicine)	(46)
Lokern	LOK	USA	Cattle, goats, sheep	Mosquitoes (anopheline, culicine), Culicoid flies	(28, 46)
Macaua		South America	Rodents, bats	Mosquitoes (culicine)	(46)
Maguari	MAG	*Aedes scapularis*, Ecuador, 1974	Sentinel animal, cattle	Mosquitoes (anopheline, culicine)	(28, 34, 46)
Main drain	MD	USA	Equine	Mosquitoes (culicine), Culicoid flies	(28, 46)
Mboke		Africa	Not known	Mosquitoes (culicine)	(46)
Ngari		*Aedes simpsoni*, Senegal, 1979	Humans	Mosquitoes (anopheline, culicine), ticks	(31, 46, 50, 51)
Northway	NOR	*Aedes* spp., Alaska, 1971	Rodents, sentinel animals	Mosquitoes (culicine)	(28, 34)
Playas		South America	Not known	Mosquitoes (culicine)	(52)
Potosi		*Aedes albopictus*, Missouri, 1989	Not known	Mosquitoes	(34, 52)
Santa Rosa		North America	Not known	Mosquitoes (culicine)	(28)
Shokwe		*Aedes cumminsii*, South Africa, 1962	Humans, rodents	Mosquitoes (anopheline, culicine)	(28, 34, 46)
Sororoca	SOR	Brazil	Not known	Mosquitoes (culicine)	(28, 46)
Tensaw	TEN	USA	Rodents, sentinel animals, cattle	Mosquitoes (anopheline, culicine)	(46)
Tlacotalpan	TLA	Mexico	Not known	Mosquitoes	(28, 46)
Tucunduba		Brazil	Not known	Mosquitoes	(28, 46)
Wyeomyia	WYO	*Wyeomyia melanocephala*, Colombia, 1940	Not known	Mosquitoes (anopheline, culicine)	(28, 34, 46)
Xingu		Human, Brazil, unknown date	Humans	Mosquitoes	(34, 52)

The taxonomy of Bunyaviruses has been challenging, given the propensity for reassortment, their general lack of characterization, and the broad geographic ranges of some genera. Often, taxonomy is first developed based on antigenic relationships, but is becoming more molecular-based as genomic methods become cheaper and data becomes available (53). Recently, the International Committee on Taxonomy of Viruses proposed an alternate classification system (54), whereby the family of Bunyavirus was suggested to become a new order Bunyavirales. *Orthobunyaviruses* would then be classified into the new family Peribunyaviridae and *Orthobunyavirus* would remain the genus. Further, Ngari, Bunyamwera, and Batai would no longer be considered separate species, but sub-types of the single species “Bunyamwera orthobunyavirus.” This method of classification was not suggested for Flaviviruses. A similar classification of Flaviviruses would have West Nile virus (WNV), dengue virus, and Yellow Fever all become sub-species when these viruses have very different geographic ranges, host ranges, clinical presentation, and within vector dynamics. As Bunyaviruses are often less characterized than Flaviviruses, there is not enough phenotypic data available to determine whether the grouping of many viruses as a single species “Bunyamwera orthobunyavirus” would be appropriate, and further efforts should be made to characterize these viruses to support taxonomic classification methods.

### Genomic Characteristics

As with all Bunyaviruses, BUNV is a segmented negative sense RNA virus. As stated, the genome is composed of three segments S, M, and L. Each segment is transcribed individually to give a single mRNA. Although there are differences in the sizes of the segments among different species and among strains within species, the averages for *Orthobunyaviruses* are 6.9 kb for L segment, 4.5 kb for M segment, and 1.0 kb for S segment (55, 56).

The S segment encodes the N (nucleocapsid) protein and a NSs, which are translated from overlapping open reading frame in the same mRNA by leaky ribosomal scanning (6, 55, 57, 58). The M mRNA encodes a polyprotein that is post-translationally cleaved by host proteases into NS, and glycoproteins (Gn and Gc) (6, 59, 60), and the L mRNA encodes the RNA-dependent RNA polymerase (55, 58, 61). As in other Bunyaviruses genera (*Nairovirus* and *Phlebovirus*), NSs and NSm have been shown to be closely related to the pathogenicity and propagation the viruses in vertebrate cells (53, 62). Although NSs is encoded by all three viruses, the length of this gene can vary among the genus (55).

The coding sequences are generally less conserved than the untranslated regions (UTRs). The functional promoter is formed by the 5′ and 3′ UTRs of each segment (55, 63–65). 3′ and 5′ UTRs are complementary (55). The deletion of internal sequences in the UTRs of BUNV resulted in attenuation of virus replication and loss of cytopathogenicity in mammalian cell culture, but the mechanistic basis of this remains unclear (55, 66).

Like other *Orthobunyaviruses*, Bunyamwera serogroup viruses are able to increase genetic and phenotypic diversity through reassortment of genome segments during mixed infections with viruses of the same group (7, 67). For example, reassortment between Bunyamwera, Maguari, Batai, and Northway viruses were obtained in cell cultures (68). NRIV is a naturally occurring reassortment resulting from a BUNV and BATV co-infection, though it is unknown whether this co-infection was in the vector or a vertebrate host (3, 31). The L and S segments of NRIV are from BUNV and the M segment from BATV (S_BUNV_, M_BATV_, L_BUNV_) (31). There are 93%, 97–98%, and 89–95% nucleotide sequences homologies respectively between BUNV and NRIV S segments; BUNV and NRIV L segments; and between BATV and NRIV M segments (1, 3). Little is known about the roles of different factors related to the occurrence of the reassortment events, such as host species, viral titers, and viral strains.

Indeed, the lack of understanding of the genomic variability of these viruses, especially NRIV, means that we are unable to really understand the phylogenetic relationships among species and strains of species, and thus we cannot fully appreciate the processes that promote reassortant and the extent of propagation of such strains. Similar challenges exist for related Bunyaviruses, such as *Nairovirus*, and recent efforts have established new species and that there is some plasticity to classification of Bunyaviruses related in large part to the lack of comprehensive sequence data (53).

### Replication in Host Cells

Bunyaviridae are enveloped viruses that replicate in the cytoplasm of mammalian cells and bud into the Golgi apparatus (68, 69). Host cell entrance is by clathrin-dependent endocytosis and vacuolar acidification, though the receptors, cellular factors, and specific pathways are not well understood (11, 70–73). The two envelope glycoproteins Gc and Gn are responsible for viral attachment and acid-activated penetration (74). It has been reported that the entry of Germiston and LaCrosse viruses, two other *Orthobunyaviruses*, is promoted by DC-SIGN (Dendritic cell-specific intracellular adhesion molecule-3-grabbing non integrin) receptors, which is seen in other Bunyaviruses, like the Phleboviruses (75). These receptors are found in dermal dendritic cells and may play a role in viral entry into mammalian cells following transmission by an arthropod bite (62, 74).

As with many arboviruses, *in vitro* infection is generally lytic in mammalian cell lines, while these viruses produce a persistent infection in mosquito cells with a lack of cytopathology (18, 76). *In vitro* infection of *Aedes aegypti* cells with BUNV revealed that NSs are essential for viral replication, as these cells were refractory to infection with a recombinant NSs gene deletion strain (rBUNdelNSs2) (18, 76). Further infections of *Aedes albopictus* cell lines with both a wild-type BUNV and another recombinant NSs gene deletion strain showed that the wild-type strain demonstrated greater fitness with 100-fold higher titers (18). In mammalian cell lines, NSs are also implicated in pathogenesis, as NSs induced shut-off of host protein synthesis and resulted in cell death (2, 18, 24).

Infection of BHK cells first showed cytopathic effect at 3 h post-infection. Using Vero cells, most BUNV group members produce clear plaques within 4 days (33). BUNV isolates were shown to grow to higher titers than NRIV isolates (11). Additionally, after serial passage on Vero cells, NRIV was found to have total conservation of N and NSs proteins, while nucleotide substitutions were observed in both the S and M segments of subsequent passages of BUNV, which may suggest that the NRIV genome is more stable in Vero cells than its parent BUNV (11).

Persistent infection in C6/36 cells is not associated with cytopathic effects and can last indefinitely. Persistent infection in mammalian cells or long-term infection in mammalian cells or culture media without cells has been noted in other Bunyaviridae viruses, but has not been reported for *Orthobunyaviruses* (69, 77).

## Transmission

Bunyamwera virus is likely maintained in nature by blood-feeding mosquitoes and susceptible vertebrate hosts. Evidence suggests *Ae. aegypti* might be the primary mosquito vector (2, 50). Experimental studies showed that *Ae. aegypti* was competent to transmit BUNV, but not a competent vector for NRIV. However, *Anopheles gambiae Giles* was competent for both viruses, while *Culex quinquefasciatus* did not demonstrate competence to transmit any of the two viruses (50). The two identified NRIV outbreaks of human febrile illnesses (Sudan in 1988 and Somalia-Kenya in 1997–1998) coincided with episodes of unusually heavy rains and extensive flooding in areas normally arid (1). This seasonal emergence pattern resembles that of RVFV, the primary vector of which is *Ae. aegypti* (21, 78).

Ngari virus has been isolated in many mosquito vectors, such as *Aedes argenteopunctatus, Aedes minutus, Aedes vexans, Aedes mcintoshi, Anopheles coustani, Aedes neoafricanus, Aedes simpsoni, Aedes vittatus, Anopheles pretoriensis, Anopheles pharoensis, Anopheles mascarensis, Culex bitaeniorhynchus, Culex tritaeniorhynchus, Culex antennatus*, and *Culex poicilipes* in Senegal in study period 1991–1994 (31). These successful isolations suggest a large vector range, which could indicate the potential for widespread geographic distributions as well as potential vertebrate host ranges, given the diversity of feeding preferences of these mosquitoes.

Batai virus has been isolated from cattle and human patients (16, 24, 34). BATV has been isolated from several mosquito species as described above, and additionally in *Anopheles claviger, Coquillettidia richiardii, Culex pipiens s.i., Ochlerotatus punctor, Ochlerotatus Communis*, and *Ae. vexans* (72, 79, 80). BATV has also been shown to be transmitted by biting midges (*Culicoides* spp.) and ticks (81).

Because risk for BATV transmission has been correlated with migratory and resident bird population distributions, BATV is thought to be associated with bird-mosquito enzootic cycle similar to WNV, Usutu virus, and Sindbis virus (72, 82). Indeed, the migratory patterns of competent bird species likely accounts for its wide geographic distribution across Europe and Asia (79, 83). BATV infection is less severe in humans, though it occurred epidemically in Scandinavia in the 1960s. In addition, neutralizing antibodies have been detected in a number of cows and one farmer on coastal farms in Finland (15, 84, 85).

## Associated Disease

All three viruses (BUNV, NRIV, and BATV) have been reported to infect humans and other mammals. BUNV causes mild symptoms in humans, such as fever, headache, joint pain, and rash. Symptomatic infections are more often reported in children, and immunocomprised patients may progress to severe encephalitis (12). In domestic animals, especially ruminants, infection leads to severe symptoms, such as spontaneous abortion and teratogenic effects (14). In 2013, BUNV was isolated for the first time in horses in Argentina after two horses developed neurological signs and died (2). CVV is endemic to North America and causes very severe diseases in humans and ruminants characterized by embryonic and fetal death, stillbirths, and multiple congenital malformations especially in ruminants. Humans are rarely infected by CVV, however, severe headache, nausea, vomiting, fatigue, encephalitis, and multiorgan failure have been reported in patients (86–88).

Batai virus infections in livestock results in abortions, premature births, and congenital defects (14, 16, 81). In humans, BATV infection has been characterized as a non-descript febrile illness in Africa and Asia and associated with an influenza-like illness in Europe (72). Febrile disease, bronchopneumonia, exudative pleurisy, catarrhal and follicular tonsillitis, and acute gastritis have all been reported clinical signs associated with BATV infection in humans (15, 89).

Ngari virus is reported to cause severe and fatal hemorrhagic fever in humans (3). NRIV clinical manifestations are similar to those of RVFV, as seen in the Kenya and Somalia outbreaks (1998–1999) and the Mauritania outbreak (2010) (4, 17). NRIV has also been isolated from small ruminants with hemorrhagic manifestations (17).

## One Health Implications of BUNV, BATV, and NRIV

While BATV is less associated with severe human disease, it can lead to significant economic losses as it infects agriculturally important mammals and bird species (14–16, 81, 89). However, given that RVFV, BUNV, and NRIV present many similarities in their clinical manifestations, co-circulate within the same vector and/or vertebrate host ranges, and share the same ecological distribution, NRIV and BUNV might contribute to outbreaks of hemorrhagic fever in these regions of both cattle and humans. Indeed, there is evidence for co-circulation in Kenya (1997–1998), where RVFV and NRIV were found in respectively 23 and 27% of human hemorrhagic fever cases tested, which was the first report of BUNV causing human disease (4). In Mauritania (2010), during an ongoing RVFV outbreak in livestock, 163 serum samples (62 from camels, 8 from cattle, and 93 from small ruminants) were tested for NRIV, and two goat samples were positive for NRIV (32). In addition, one of these two samples was also IgM positive for RVFV, suggesting a recent (possibly co-) infection of RVFV (32). These data suggest that in areas, where RVFV is reported but not confirmed or where the etiologic agent has not been molecularly confirmed, BUNV and NRIV should be included in a differential diagnostic panel for hemorrhagic fevers of humans and animals (1, 3).

That being said, the diagnostic capabilities available for *Orthobunyaviruses* in general are limited. Detection of nucleic acid has been utilized and is capable of differentiating between BATV, NRIV, and BUNV, but requires multiple genes to be amplified. And inherent in this method is the assumption that there are no co-infections of NRIV and one of its parental viruses, as theoretically, a co-infection of BATV and BUNV would give the same pcr read-out as NRIV. Thus, serological assays are needed to detect circulation of these viruses. Cross-neutralization among these viruses is not limited to these three, and traditional PRNT methods are likely to be unable to distinguish to the species level (28). In addition, there is a commercially available nucleotide and antibody bundle that was developed for Cache Valley Fever virus (Discovery Antibodies, UK), a strain of BUNV, which purports to cross-react with BATV. Thus, while diagnostic capability for these three *Orthobunyaviruses* exists, whether or not there is an ability to definitively identify which virus is the agent remains unclear.

In addition to diagnostic capabilities, there is a better need to understand control and prevention of transmission. A first step in this process is identifying the mosquito vectors of medical and veterinary importance. To that point, the geographic distribution(s) of these three viruses should be better characterized in effort to accurately assess which mosquito vectors could transmit. Additionally, comparative vector competence studies should be conducted in efforts to elucidate the relative transmission potential of these three viruses. Although BATV is mainly defined as European/Asian virus and has only once been isolated in Africa, it may be present in several other countries in Africa, but underreported, since NRIV has only been isolated in Africa and BATV is its presumptive parent. After identification of likely vectors, appropriate control methods can be implemented, such as additional spraying efforts and behavior modification and education programs. And since the life cycles of vectors are closely related to climatic conditions, the role of climate change in the continued circulation, the potential for altered and expanded geographic distributions, and seasonality of transmission should be considered in all prevention campaigns.

## Conclusion

The similarity between these viruses suggests that differential diagnosis of febrile and/or hemorrhagic diseases should be done carefully in areas where these viruses are likely underreported. The overlap in distribution, vertebrate, and vector hosts, and similarity in clinical presentation makes clinical identification of disease agents difficult. Since NRIV has been isolated twice during concurrent RVFV outbreaks, the co-circulation of these two viruses and their respective contributions to the burden of human and animal disease needs to be better understood. These viruses pose potential threats to animal health, food security, and human public health. Thus, a One Health approach to the promotion of understanding these viruses should be undertaken to define geographic risk regions, vector control strategies, and diagnostic development. Overall, the relevance of these and other lesser-characterized Bunyaviruses can only be appropriately prioritized as these viruses are comprehensively studied.

## Author Contributions

All authors contributed equally to the conception of this review. MD and RC executed a first draft. All authors contributed equally to the final draft and editing of the manuscript.

## Conflict of Interest Statement

The authors declare that the research was conducted in the absence of any commercial or financial relationships that could be construed as a potential conflict of interest. The reviewer KD and handling Editor declared their shared affiliation.

## Supplementary Material

Table S1 Geographic locations, coordinates, and year of report of three *Orthobunyaviruses*: Bunyamwera (BUNV), Bataie (BATV), and Ngari (NRIV).
